# Traditional Versus Velocity-Based Resistance Training in Competitive Female Cyclists: A Randomized Controlled Trial

**DOI:** 10.3389/fphys.2021.586113

**Published:** 2021-02-25

**Authors:** Almudena Montalvo-Pérez, Lidia B. Alejo, Pedro L. Valenzuela, Jaime Gil-Cabrera, Eduardo Talavera, Alejandro Lucia, David Barranco-Gil

**Affiliations:** ^1^Faculty of Sport Sciences, Universidad Europea de Madrid, Madrid, Spain; ^2^Instituto de Investigación Hospital 12 de Octubre (imas12), Madrid, Spain; ^3^Department of Systems Biology, University of Alcalá, Madrid, Spain; ^4^Department of Sport and Health, Spanish Agency for Health Protection in Sport (AEPSAD), Madrid, Spain

**Keywords:** performance, strength, endurance, cycling, female, power

## Abstract

We assessed the effects of a short-term velocity-based resistance training (VBRT, where exercise intensity is individualized based on the loads and repetitions that maximize power output) program compared with traditional resistance training (TRT, where the same number of repetitions and relative load are used for every individual) on body composition, muscle strength/power, and endurance performance in competitive female cyclists. Seventeen participants were randomly assigned to 6 weeks (two sessions/week) of TRT (*n* = 8) or VBRT (*n* = 9), during which they maintained their usual endurance program. Both interventions included squat, hip thrust, and split squat exercises. Training loads were continuously registered, and outcomes were measures of muscle strength/power, body composition, and endurance performance (incremental test and 8-min time trial). No differences between TRT and VBRT groups were found for overall internal training loads during resistance training or cycling sessions (*p* > 0.05). Both interventions led to significant improvements in all strength/power-related outcomes, but VBRT induced greater improvements than TRT in maximum muscle strength and power as assessed with the hip thrust exercise (*p* < 0.05 for the group by time interaction effect). However, no significant group by time interaction effect was found for body composition or endurance performance-related outcomes. In conclusion, the addition of a short-term intervention of VBRT or TRT to the usual training regimen of competitive female cyclists improves muscle strength/power, albeit VBRT might induce superior gains on maximum strength/power for the hip thrust exercise.

## Introduction

Strong evidence supports the benefits of resistance (“strength”) training for endurance athletes (Beattie et al., 2014; Rønnestad and Mujika, 2014; Blagrove et al., 2018). Besides the expected improvements in muscle strength, resistance training has been reported to increase sprint ability as well as endurance performance indicators in cyclists (Rønnestad et al., 2010, 2017; Sunde et al., 2010; Beattie et al., 2017; Kristoffersen et al., 2019). Nevertheless, most research in the field has been conducted in male cyclists, with only scarcer data available for female cyclists (Bishop et al., 1999; Vikmoen et al., 2016, 2017, 2020). A classic study by Bishop et al. (1999) reported no changes in endurance performance in trained female cyclists after a 12-week resistance training program that consisted of parallel squats performed to failure with a load corresponding to 2–8 repetition maximum (RM). However, more recent studies have shown improvements in muscle strength and cycling economy/performance after an 11-week resistance training intervention (3 × 4–10 RM) in female cyclists (Vikmoen et al., 2016, 2017, 2020).

New resistance training modalities are gaining popularity in recent years, notably velocity-based resistance training (VBRT) (Guerriero et al., 2018; Weakley et al., 2020). As opposed to “traditional” resistance training (TRT), in VBRT, exercise intensity is not individualized based on a given percentage of RM but on the velocity at which loads can be lifted (González-Badillo and Sánchez-Medina, 2010). Moreover, the number of repetitions performed depends on the magnitude of velocity loss during the set, which would enable a stable force–velocity relationship (i.e., lower loss of velocity for a given load) regardless of individual relative strength and a better control of fatigue (Sánchez-Medina and González-Badillo, 2011). VBRT might therefore reduce unnecessary mechanical stress and increase the number of repetitions performed at high velocities compared to TRT (Banyard et al., 2019). Further, Orange et al. (2019) and Dorrell et al. (2020) recently reported that VBRT could be more effective to improve muscle strength and/or power than TRT despite requiring a lower total training volume. In the same line, a 4-week VBRT program was recently reported to provide similar muscle strength gains in men and women compared to TRT, but mechanical and perceived stress were lower with the former (Pelka and Claytor, 2019). Thus, VBRT appears as a potentially superior – or at least a more efficient for a given total training volume – strategy than TRT for the enhancement of muscle strength and body composition.

Optimum load training (OPT) is a commonly used type of VBRT that consists of training with the individual load at which the highest power output is achieved, usually known as “optimum power load” (OPL) (Rauch et al., 2018). OPT has been shown to improve muscle strength and power, as well as body composition (Rauch et al., 2018; Ribeiro et al., 2020). Notably, evidence suggests that VBRT, and particularly OPT, might be superior to TRT for the improvement of sprint performance (Ribeiro et al., 2020). For instance, Loturco et al. (2016) observed that OPT induced greater improvements in sprinting and jumping capacity than TRT in soccer players. In turn, Ribeiro et al. recently reported that OPT was superior to classic plyometric training for improving change of direction performance and sprinting ability in young soccer players. Scarce evidence exists, however, on how VBRT compares to TRT regarding potential improvements in endurance performance. We recently found that VBRT and TRT induced similar improvements in body composition, muscle strength/power, and endurance performance indicators in male elite cyclists (Gil Cabrera et al., 2020). In another recent study, we observed similar improvements in endurance-related parameters with VBRT and cycling-based “power training” (i.e., short sprint training) in male elite cyclists, although VBRT tended to induce greater benefits with regard to body composition and muscle strength/power (Valenzuela et al., 2020).

Evidence on the comparison between VBRT and TRT is therefore still scarce and, to our knowledge, no previous study has assessed the effects of VBRT on female cyclists’ performance. In this context, the aim of this study was to compare the effects of 6 weeks of TRT or VBRT on body composition, muscle strength/power, and endurance performance-related outcomes in female elite cyclists. Our hypothesis was that VBRT would induce greater – or at least similar – benefits in endurance performance and body composition compared to TRT.

## Materials and Methods

### Experimental Approach to the Problem

The study was conducted during the precompetitive season (October 2019 to December 2019), after 1 month of preseason training that included endurance and light-load resistance training. Participants were paired-matched based on their maximal oxygen uptake (VO_2__max_, see below for a description of the assessment method) at baseline. Thus, among each pair of cyclists with a similar baseline VO_2__max_, one was randomly assigned to a TRT group (*n* = 8), and the other to a VBRT group (*n* = 9) (Decroix et al., 2016). Both TRT and VBRT interventions lasted 6 weeks (two sessions/week). Two weeks before the beginning of the study, both groups performed a familiarization phase of four sessions with the TRT/VBRT exercises. A minimum adherence of 90% to sessions was deemed necessary for cyclists’ data to be included in the study.

### Subjects

A group of female cyclists (*n* = 17, age 26 ± 7 years, VO_2__max_ 55 ± 5.8 ml⋅kg^–1^⋅min^–1^) volunteered to participate in the study. Inclusion criteria were age > 18 years, competing at national or international level [i.e., level 4, or “well/highly trained or competitive” according to the classification proposed by Decroix et al. (2016)] and being free of musculoskeletal injuries or other conditions that could hinder their participation. Among 57 potentially eligible cyclists in the city of Madrid, 17 volunteered to participate in the study, which was superimposed to their usual cycling training program. Participants had previous experience with TRT (>2 years, 1 or 2 sessions/week) but not with VBRT specifically.

All participants had the procedures explained and provided written informed consent. The study was approved by the Ethical Committee of the Fuenlabrada University Hospital (approval number 19/86).

### Procedures

Outcomes were assessed the week before and after the 6-week intervention, respectively. In both pre- and post-intervention assessment periods, participants attended the laboratory facilities on three different days (at approximately the same time of the day and interspersed by 48 h), where they underwent: body composition assessment and maximal incremental tests (first testing session), strength tests (second testing session), and a simulated time trial (third testing session) (see below for further details). Subjects were instructed to maintain their normal dietary pattern and to refrain from doing intense exercise and consuming ergogenic aids/caffeine 48 h before each testing session. A questionnaire regarding menstrual cycle disturbances was filled out by all the participants.

### Outcomes Follow

Body mass was measured using a digital scale (Seca 784, Hamburg, Germany). Body composition [whole body fat and muscle mass, and bone mineral density (BMD) and content (BMC)] was evaluated by dual energy X-ray absorptiometry (DXA, Hologic QDR series Discovery; Bedford, MA). DXA assessments were performed at least 2 days after the last exercise session. Participants were encouraged to maintain a similar sleeping and eating schedule the day before each testing session and were advised to attend the laboratory in a euhydrated state.

All endurance performance-related outcomes were assessed with the participants’ own bicycle attached to a validated indoor trainer (Hammer, CycleOps, Madison, WI) (Lillo-Bevia and Pallarés, 2018). After a standardized 10-min warm-up at 75 W, participants performed a maximal incremental cycling test. The test started at 75 W and workload was increased following a ramp-like protocol [i.e., 5-W increases every 12 s (average = 25 W/min)]. Gas exchange data were collected breath-by-breath (Ultima Series Medgraphics; Cardiorespiratory Diagnostics, Saint Paul, MN). The tests were concluded when participants reached volitional exhaustion or when they could no longer maintain cadence at ≥ 70 rpm. The ventilatory threshold (VT) was determined through visual inspection as the workload at which an increase in both the ventilatory equivalent for oxygen (VE⋅VO_2_^–1^) and end-tidal partial pressure of carbon dioxide (PetCO_2_) occurred with no concomitant increase in the ventilatory equivalent for carbon dioxide (VE⋅VCO_2_^–1^), whereas the respiratory compensation threshold (RCP, also termed “second ventilatory threshold”) corresponded to the work rate at which both VE⋅VO_2_^–1^ and VE⋅VCO_2_^–1^ increased together with a decrease in PetCO_2_ (Lucía et al., 2000). Peak power output (PPO) was defined as the highest power output (PO) value reached during the test, and VO_2__max_ was defined as the highest VO_2_ value (mean of 30 s) attained during the test (Gil Cabrera et al., 2020).

In the second visit, muscle strength and power-related outcomes were assessed with the same equipment as that used for all TRT/VBRT sessions. Incremental loading tests for the squat, lunge, and hip-thrust exercises were performed in a randomized order on a Smith machine (Signature Series, Life Fitness, IL, United States), and mean propulsive velocity (MPV) and power (MPP) of the bar during the concentric phase were measured with a validated linear position transducer (T-Force System; Ergotech, Murcia, Spain) (Sánchez-Medina and González-Badillo, 2011). The initial weight was 20 kg (i.e., only the bar), and the load was increased by 5–10 kg until a decrease in MPP was observed in two consecutive loads. Participants performed three consecutive repetitions with each load, and a 3-min rest was allowed between loads. The highest MPP registered for each exercise was used for analysis.

After participants rested for 10 min upon completion of the incremental loading “strength” test, we assessed the number of repetitions that they could perform with their MPP-associated load (i.e., OPL) before attaining <90% of their MPP during the sets, which was used for training prescription (see more information further below) (Sarabia et al., 2017). We also estimated the 1RM for each exercise based on the equations proposed elsewhere for squat (Conceição et al., 2016) and hip thrust exercises (de Hoyo et al., 2019). In the case of the lunge exercise – for which no validated equations are available – we used the same procedure as for the squat exercise, following the methodology used in previous studies (Conceição et al., 2016; Gil Cabrera et al., 2020; Valenzuela et al., 2020).

On a separate session and at least 48 h after the incremental test, participants performed a simulated 8-min time trial after a standardized 10-min warm-up at 60% of their PPO. The mean PO [in both absolute values (W) or relative to body mass (W⋅kg^–1^)] was registered during each time trial. Participants were instructed to attain the highest mean PO possible, but they received no instructions regarding pacing and were blinded to PO values during the trial. Performance in this test has been proven to be reliable (intra-class correlation coefficient = 0.93) and valid to measure changes in fitness (Klika et al., 2007), as well as to be strongly correlated with other laboratory-based predictors of endurance performance, such as the PO eliciting a blood lactate concentration of 4 mM⋅L^–1^ (Sanders et al., 2020).

### Intervention

All participants performed two TRT or VBRT sessions/week (from Monday to Friday) for a total of 6 weeks. Resistance training sessions were interspersed by a minimum of 48 h, with a recovery period of at least 8 h elapsed between cycling and resistance training sessions (Berryman et al., 2019). All the sessions were supervised by a fitness specialist and included squat, hip thrust, and lunge exercises, which were performed in varying order during the study.

The TRT program was prescribed following the recommendations proposed elsewhere (Rønnestad and Mujika, 2014; Mujika et al., 2016). Thus, participants performed three sets per exercise (interspersed by 120-s rest periods), with intensity progressively increasing from 80 to 90% of 1RM from the start to the end of the intervention period, whereas number of repetitions decreased from 8 to 4 (Table 1). The same number of repetitions and relative load (% of 1RM) was used in all three exercises at each phase.

**TABLE 1:** Characteristics of the traditional (TRT) and velocity-based resistance training (VBRT) interventions.

Intervention	Variable	Weeks 1–2		Weeks 3–6	
		Day 1	Day 2	Day 1	Day 2
	
		Exercises: squat, hip thrust, and split squat			
TRT	Training (sets × repetitions)	3 × 8	3 × 5	3 × 6	3 × 4
	Load (% of 1RM)	80%	87%	85%	90%
	Rest between sets (s)	120			
VBRT	Training (sets × repetitions)	3 × maximum number or repetitions at > 90% of OPL (8 ± 3 repetitions)			
	Load (% of 1RM)	OPL (65 ± 10%)			
	Rest between sets (s)	120			

*OPL, optimum power load; RM, repetition maximum.*

Participants in the VBRT group also performed three sets per exercise interspersed by 120-s rest periods, but the load was individualized and each participant trained with their MPP-associated load (i.e., OPL) for each specific exercise, as explained elsewhere (Sarabia et al., 2017; Gil Cabrera et al., 2020; Valenzuela et al., 2020). The OPL and the number of repetitions were determined for each participant during baseline tests. During each set, participants performed as many repetitions as possible before 90% of the MPP was attained. Thus, the average load lifted and the number of repetitions per set varied between participants (averaging 65 ± 10% of 1RM and 8 ± 3 repetitions, respectively).

The weight lifted (computed as number of repetitions performed multiplied by the load lifted, in kg) during each TRT or VBRT session was recorded as a measure of external training load. We also analyzed total internal training loads {computed as training volume [session length, in minutes] multiplied by the rating of perceived exertion [RPE, using the 1–10 Borg scale (Borg, 1998)]} during all TRT/VBRT and cycling sessions, respectively. This variable has been proven to be a valid and reliable marker of internal training load, being strongly correlated with other markers of external training load (Arney et al., 2019; van Erp et al., 2019).

### Statistical Analysis

Data are shown as mean ± standard deviation (SD). We compared both types of interventions using a two-factor [group (TRT, VBRT)] analysis of variance (ANOVA) with repeated measures on time (baseline, post-intervention), and with *post hoc* analyses done with the Bonferroni test. In order to minimize type I error, between-group analyses were conducted only when a significant group by time interaction was found. Effect sizes for between-group differences at baseline (Hedges’ *g*) as well as for between-group differences in intervention effects [partial eta-squared ($\eta_{p}^{2}$)] were calculated and considered small, moderate, or large (*g* > 0.2, > 0.5, or > 0.8, respectively; and η_*p*_^2^ > 0.01, >0.06, or >0.14, respectively) (Hopkins et al., 2009; Cohen, 2013). All analyses were performed using the SPSS statistical package (version 23.0, IBM statistics, Chicago, IL) with α = 0.05.

## Results

No between-group differences were found for baseline descriptive variables except for age (30 ± 5 and 22 ± 7 years for TRT and VBRT group, respectively, *p* = 0.027): body mass (58.3 ± 6.1 and 57.8 ± 6.9 kg, *p* = 0.815), height (169 ± 5 and 165 ± 7 cm, *p* = 0.114), VO_2__max_ (54.6 ± 5.2 and 55.4 ± 6.7 ml⋅kg^–1^⋅min^–1^, *p* = 0.815), and PPO (288 ± 47 and 291 ± 33 W, *p* = 0.370, or 4.79 ± 0.54 and 4.97 ± 0.34 W⋅kg^–1^, *p* = 0.236). Moreover, no significant differences were found at baseline for performance outcomes, including muscle strength/power (all *p* values > 0.05 and in fact all >0.20). Three cyclists in TRT suffered menstrual cycle disorders (one amenorrhea and two dysmenorrhea) vs. four in VBRT (two amenorrhea and two dysmenorrhea). All participants completed the study with an adherence to the intervention sessions of 100%, and their data were therefore included in the analyses. No injuries or training-related adverse events were reported.

### Training Loads

Both groups performed the same number of resistance training [median (interquartile range) of 12 (0) and 12 (0) for TRT and VBRT, respectively, *p* = 1.000] and outdoor cycling sessions [27 (16) and 24 (10), *p* = 0.440].

**FIGURE 1:**
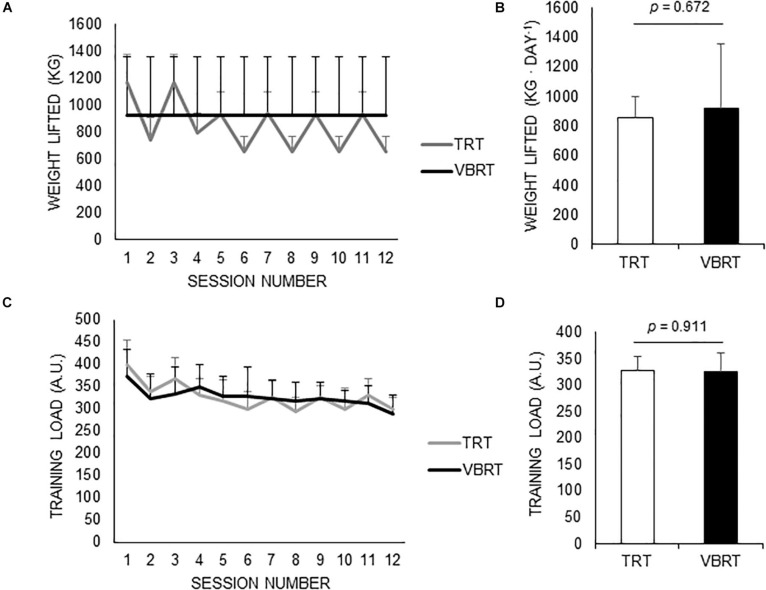
Training loads by group. Total weight lifted in kg **(A)** or in kg⋅day^–1^ **(B)** during the traditional (TRT) or velocity-based resistance training (VBRT) sessions. Total internal training loads [in arbitrary units (A.U.)] by group across TRT/VBRT sessions **(C)** and during outdoor cycling sessions **(D)**. TRT, traditional resistance training; VBRT, velocity-based resistance training.

A significant time (*p* < 0.001) and group by time interaction effect (*p* < 0.001, η_*p*_^2^ = 0.91) was found for the total weight lifted during the TRT or VBRT sessions, but with no *post hoc* differences (Figure 1A). Likewise, no differences were found between groups for the average weight lifted during the resistance training sessions (Figure 1B). A significant time effect (*p* < 0.001) but no significant group by time interaction effect (*p* = 0.181) was found for total internal training loads during TRT/VBRT sessions (Figure 1C). In turn, no significant time (*p* = 0.391) or group by time interaction effect (*p* = 0.196) was found for total internal training loads during cycling sessions (Figure 1D). On the other hand, TRT resulted in a higher average exercise intensity for all exercises in resistance training sessions (squat: 85.5 ± 0.7% vs. 68.0 ± 4.0% of 1RM, *p* < 0.001; hip thrust: 85.9 ± 0.8% vs. 57.3 ± 10.7% of 1RM, *p* < 0.001; lunge: 85.8 ± 0.7% vs. 68.3 ± 4.1% of 1RM, *p* < 0.001).

### Body Composition

A significant time effect (*p* = 0.019) was observed for body mass. However, no significant within-group differences were found from baseline to post-intervention, and no time or group by time interaction effect was found for this or the rest of body composition-related outcomes (all *p* > 0.05, Table 2).

**TABLE 2:** Results of body composition.

Outcome	Group	Baseline	Effect size for between-group differences at baseline (Hedges’ *g*)	Post-intervention	Within-group comparison (baseline vs. post-intervention) *p* value	Time effect, *p* value	Group by time interaction effect, *p* value	Effect size for between-group differences in intervention effects (η_*p*_^2^)
Body mass (kg)	TRT	58.3 ± 6.1	0.073	59.4 ± 7.0	0.079	**0.019**	0.920	0.001
	VBRT	57.8 ± 6.9		58.8 ± 6.6	0.084			
Fat mass (kg)	TRT	16.6 ± 4.4	0.355	17.1 ± 4.0	0.198	0.612	0.170	0.122
	VBRT	15.3 ± 2.4		15.0 ± 2.6	0.511			
Muscle mass (kg)	TRT	39.5 ± 4.8	0.151	40.0 ± 6.1	0.398	0.058	0.446	0.039
	VBRT	40.3 ± 5.2		41.5 ± 5.4	0.057			
BMD (g cm^–2^)	TRT	1.15 ± 0.10	0.368	1.14 ± 0.10	0.345	0.838	0.245	0.089
	VBRT	1.18 ± 0.05		1.18 ± 0.05	0.477			
BMC (g)	TRT	2.24 ± 0.41	0.078	2.24 ± 0.39	0.849	0.751	0.968	0.000
	VBRT	2.27 ± 0.32		2.27 ± 0.30	0.794			

*Data are mean ± SD and significant *p* values are in bold. BMC, bone mineral content; BMD, bone mineral density; TRT, traditional resistance training; VBRT, velocity-based resistance training.*

### Muscle Strength/Power

Significant within-group improvements from baseline to post-intervention were found for all outcomes in both groups (all *p* < 0.05, *g* = 0.85 to 1.59 for TRT and 0.87 to 2.86 for VBRT). A significant and large group by time interaction effect was found for hip thrust 1RM (*p* = 0.015, η_*p*_^2^ = 0.34), MMP (*p* = 0.015, η_*p*_^2^ = 0.33), and MMP relative to lower-body muscle mass (*p* = 0.042, η_*p*_^2^ = 0.25), which was due to *post hoc* significant differences at post-intervention. No significant group by time interaction effect was found for the remainder of muscle strength-related outcomes (Table 3).

**TABLE 3:** Results of muscle strength/power-related outcomes.

Outcome	Group	Baseline	Effect size for between-group differences at baseline (Hedges’ *g*)	Post-intervention	Within-group comparison (baseline vs. post-intervention) *p* value	Time effect, *p* value	Group by time interaction effect, *p* value	Effect size for between-group differences in intervention effects (η_*p*_^2^)
Squat 1RM (kg)	TRT	48 ± 13	0.373	65 ± 6	**0.001**	**<0.001**	0.990	0.000
	VBRT	54 ± 17		70 ± 12	**<0.001**			
Squat MMP (W)	TRT	285 ± 98	0.553	362 ± 71	**0.002**	**<0.001**	0.499	0.031
	VBRT	345 ± 107		442 ± 81	**<0.001**			
Squat MMP [W/lower body muscle mass (kg)]	TRT	20 ± 5	0.701	27 ± 5	**0.001**	**<0.001**	0.846	0.003
	VBRT	25 ± 8		31 ± 6	**<0.001**			
Hip thrust 1RM (kg)	TRT	62 ± 19	0.109	84 ± 15	**<0.001**	**<0.001**	**0.015**	0.336
	VBRT	60 ± 16		99 ± 9_†_	**<0.001**			
Hip thrust MMP (W)	TRT	278 ± 98	0.054	363 ± 71	**0.003**	**<0.001**	**0.015**	0.335
	VBRT	283 ± 78		459 ± 71_†_	**<0.001**			
Hip thrust MMP [W/lower body muscle mass (kg)]	TRT	20 ± 4	0.000	27 ± 4	**0.001**	**<0.001**	**0.042**	0.247
	VBRT	20 ± 6		32 ± 3_†_	**<0.001**			
Split squat 1RM (kg)	TRT	43 ± 10	0.000	59 ± 11	**0.001**	**<0.001**	0.386	0.050
	VBRT	43 ± 9		64 ± 13	**<0.001**			
Split squat MMP (W)	TRT	228 ± 74	0.503	328 ± 82	**0.007**	**<0.001**	0.590	0.020
	VBRT	264 ± 62		386 ± 95	**<0.001**			
Split squat MMP [W/lower body muscle mass (kg)]	TRT	17 ± 5	0.463	24 ± 4	**0.001**	**<0.001**	0.809	0.004
	VBRT	20 ± 7		27 ± 8	**<0.001**			

*Data are mean ± SD and significant *p* values are in bold. Symbols: †*p* < 0.05 vs. TRT at the same time point. 1RM, one-repetition maximum; MMP, maximum mean power output; TRT, traditional resistance training; VBRT, velocity-based resistance training.*

### Endurance Performance Indicators

**FIGURE 2:**
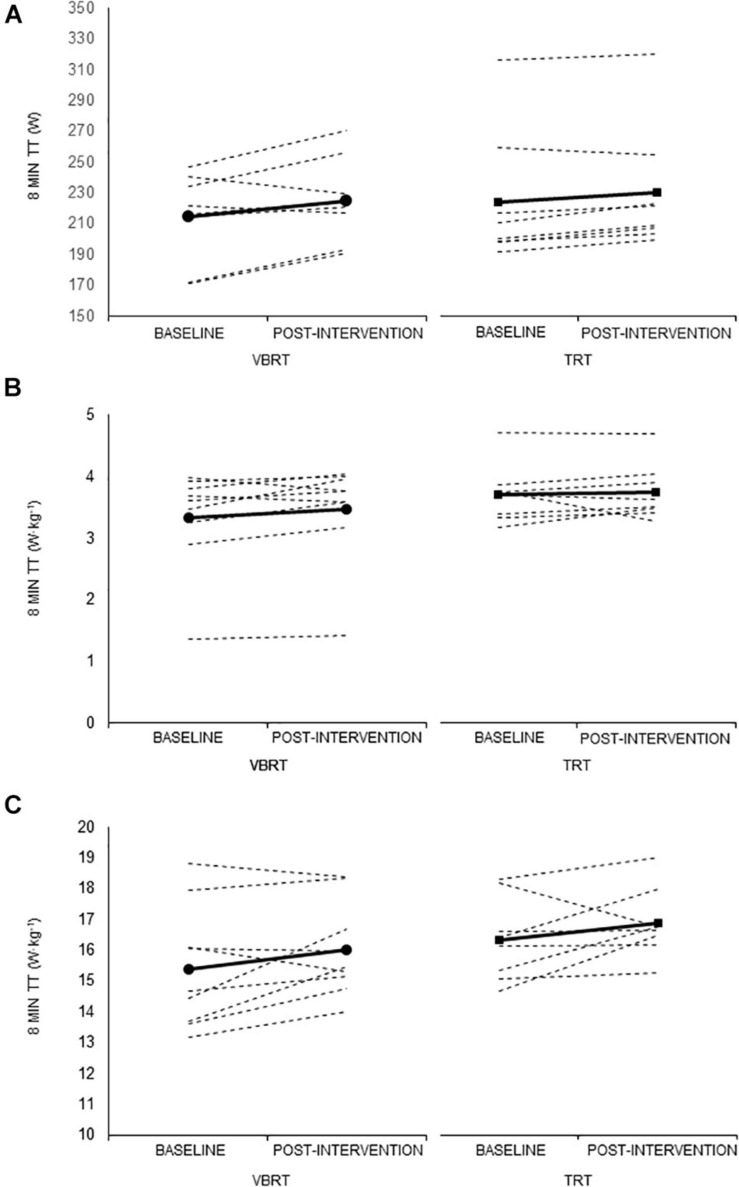
Individual data of average power output (PO) during the 8-min time trial (TT). PO is expressed in W **(A)**, W/total body mass (kg) **(B)**, and W/lower-body muscle mass (kg) **(C)**.

A significant time effect (*p* = 0.003) was found for time trial performance when expressed in absolute values (W), with significant improvements from baseline to post-intervention for the VBRT group in separate analyses (*p* = 0.006, *g* = 0.36), but not for TRT (*p* = 0.099, *g* = 0.14) (Table 4). On the other hand, a significant time effect (with improvements from baseline to post-intervention) was found in the two groups for time trial performance expressed as W⋅kg^–1^ (both *p* < 0.001). A significant time effect was also found for PPO expressed in absolute values (W, *p* = 0.050), but *post hoc* analyses revealed no significant within-group differences. Despite the aforementioned significant differences over time within groups, no significant group by time interaction effect was found for any of the analyzed endurance-related outcomes, thereby indicating that no intervention was actually superior to the other one to improve cycling performance. Individual data of time trial performance by group are shown in Figure 2.

**TABLE 4:** Results of endurance performance-related outcomes.

Outcome	Group	Baseline	Effect size for between-group differences at baseline (Hedges’ *g*)	Post-intervention	Within-group comparison (baseline vs. post-intervention) *p* value	Time effect, *p* value	Group by time interaction effect, *p* value	Effect size for between-group differences in intervention effects (η_*p*_^2^)
**VO_2__max_**								
mLO_2_/total body mass (kg)/min	TRT	54.6 ± 5.2	0.126	55.5 ± 5.2	0.571	0.545	0.827	0.003
	VBRT	55.4 ± 6.7		55.8 ± 5.5	0.776			
mLO_2_/lower-body muscle mass (kg)/min	TRT	240 ± 19	0.196	245 ± 22	0.425	0.564	0.555	0.024
	VBRT	235 ± 28		233 ± 19	0.992			
**PPO**								
W	TRT	288 ± 47	0.071	296 ± 52	0.138	**0.046**	0.913	0.001
	VBRT	291 ± 33		298 ± 36	0.154			
W/total body mass (kg)	TRT	4.8 ± 0.5	0.468	4.9 ± 0.6	0.122	0.177	0.347	0.059
	VBRT	5.0 ± 0.3		5.0 ± 0.4	0.751			
W/lower-body muscle mass (kg)	TRT	21.0 ± 1.1	0.111	21.7 ± 1.5	0.152	0.120	0.592	0.020
	VBRT	20.8 ± 2.1		21.2 ± 1.8	0.434			
**PO at the VT**								
W	TRT	163 ± 22	0.491	171 ± 22	0.340	0.165	0.978	0.000
	VBRT	154 ± 12		164 ± 28	0.295			
W/total body mass (kg)	TRT	2.7 ± 0.4	0.000	2.9 ± 0.3	0.333	0.237	0.825	0.003
	VBRT	2.7 ± 0.3		2.8 ± 0.5	0.475			
W/lower-body muscle mass (kg)	TRT	12.0 ± 1.7	0.502	12.7 ± 1.9	0.328	0.202	0.893	0.001
	VBRT	11.1 ± 1.7		11.7 ± 2.7	0.397			
**PO at the RCP**								
W	TRT	245 ± 20	0.206	249 ± 40	0.768	0.726	0.937	0.000
	VBRT	239 ± 33		242 ± 36	0.812			
W/total body mass (kg)	TRT	4.1 ± 0.5	0.000	4.2 ± 0.4	0.844	0.983	0.758	0.007
	VBRT	4.1 ± 0.6		4.1 ± 0.5	0.810			
W/lower-body muscle mass (kg)	TRT	18.1 ± 1.7	0.384	18.3 ± 1.9	0.791	0.832	0.862	0.002
	VBRT	17.2 ± 2.6		17.2 ± 2.5	0.978			
**8-min TT performance (average PO)**								
W	TRT	224 ± 43	0.241	230 ± 40	0.099	**0.003**	0.361	0.056
	VBRT	215 ± 27		225 ± 26	**0.006**			
W/total body mass (kg)	TRT	3.7 ± 0.5	0.561	3.8 ± 0.5	0.686	0.134	0.340	0.061
	VBRT	3.3 ± 0.8		3.5 ± 0.8	0.080			
W/lower-body muscle mass (kg)	TRT	13.0 ± 0.8	0.388	16.9 ± 1.1	**< 0.001**	**<0.001**	0.238	0.091
	VBRT	12.5 ± 1.5		16.0 ± 1.5	**< 0.001**			

*Data are mean ± SD and significant *p* values are in bold. PO, power output; PPO, peak power output; RCP, respiratory compensatory threshold; TRT, traditional resistance training; TT, time trial; VBRT, velocity-based resistance training; VO_2__max_, maximal oxygen uptake; VT, ventilatory threshold. An actual plateau in VO_2_ values, defined as an increase in VO_2_ values of less than 1.5 ml⋅kg^–1^⋅min^–1^ between two or more consecutive 1-min workloads in the final part of the tests (Lucía et al., 2006) was observed in 48% (baseline tests) and 54% (post-intervention tests) of the participants in the VBRT group, and in 42% (baseline) and 46% (post-intervention) of the participants in the TRT group.*

## Discussion

The main finding of this study was that a 6-week intervention of TRT or VBRT induced overall comparable improvements in muscle power/strength and in endurance performance indicators in competitive female cyclists. Some differences were however found between the two interventions, with VBRT resulting in greater gains in muscle strength and power on the hip thrust exercise from baseline to post-intervention than TRT. To our knowledge, this is the first study that assesses the effects of VBRT in female cyclists.

Besides improvements in muscle power/strength (which were overall of moderate-to-very large magnitude), we found an overall trend – of trivial-to-small magnitude – toward an improved endurance performance with both resistance training programs, although statistical significance was found only for VBRT. These findings are in close agreement with the results of the studies of Vikmoen et al. (2020), who found that a concurrent TRT and endurance training program improved 1RM on leg press, as well as 1RM on the half squat and performance during a 5-min all-out test (performed after 3 h of submaximal work) in female duathletes (Vikmoen et al., 2017). These authors have also reported improvements in lower-limb strength and performance during an all-out 40-min cycling bout with this training modality in female cyclists (Vikmoen et al., 2016). Moreover, the improvement observed on 8-min time trial performance in the present study (∼3–5%) is overall in line with that recently observed by us with TRT and VBRT in male elite cyclists (Gil Cabrera et al., 2020), and in fact greater than the average coefficient of variation reported for cycle-ergometer performance tests (<2%) (Hopkins et al., 2001). It must be noted, however, that contrary to previous studies in male cyclists (Sunde et al., 2010; Rønnestad et al., 2015), here, we found no intervention benefits for different laboratory-based physiological markers of endurance performance (e.g., RCP, VO_2__max_). On the other hand, future research is warranted to elucidate whether VBRT can improve other variables not assessed here, such as cycling economy.

Although we observed significant improvements in all markers of muscle strength/power with both interventions, VBRT led to greater improvements in strength and power for one of the three exercises performed, hip thrust, and induced a significant improvement on time trial performance (vs. no significant change with TRT). Previous evidence supports the benefits of VBRT for the improvement of muscle strength and power. Loturco et al. (2016) reported that, in professional male soccer players, training with the OPL – as we did here – resulted in greater power improvements (measured through sprints and jumping ability) than a TRT program. Rauch et al. (2018) also reported that two types of VBRT – including training with the OPL – increased muscle strength, power, and mass in female volleyball players.

It must be noted, however, that in the present study, we found no group by time intervention effect for endurance performance indicators (including time trial performance), or for muscle strength/power as assessed with exercises other than the hip thrust (squat and lunge). Similarly, we recently found that the addition of TRT or VBRT to the usual endurance training regimen of male elite cyclists during 8 weeks induced similar improvements in muscle strength/power, endurance performance, or body composition (Gil Cabrera et al., 2020). Therefore, further evidence is needed to confirm the eventual superiority of VBRT over TRT. In this regard, some studies have reported that VBRT might be a more efficient strategy than TRT, even if the same performance benefits are obtained. For instance, Dorrell et al. (2020) reported a higher training volume (6%) with TRT compared with VBRT. Likewise, Sarabia et al. (2017) observed higher RPE values and larger performance decrements during the resistance training sessions with TRT than with VBRT, and Orange et al. (2019) also observed an elevated perceived stress with the former (although no differences were found for other markers such as fatigue, soreness, or overall wellness score). Avoiding resistance training-induced muscle fatigue (e.g., muscle soreness) is of major importance for cyclists, as this condition might impair the quality of endurance training sessions and thus limit the improvement of endurance skills (Doma et al., 2017). In the present study, we observed that, although there were no differences in the total weight lifted between interventions, TRT resulted in a higher average relative training intensity (expressed as% of 1RM) than VBRT, which could potentially induce a greater fatigue. Further research is, however, needed to confirm the eventual superiority of VBRT to reduce the perceived stress of training. Moreover, given that participants in each group trained at a different relative intensity, one could hypothesize that VBRT might exert different effects on the force–velocity relationship (e.g., improving performance with lighter loads), but this should also be confirmed in future studies.

It must also be noted that the trend toward an increase in total body mass (without reaching statistical significance in within-group analyses) observed with both resistance training interventions might be viewed, at least partly, as a potentially negative adaptation in terms of actual cycling performance if not accompanied by a proportional increase in power output – at least during uphill climbing. There is indeed some reluctance among cyclists to include resistance exercises in their usual training regimen due to the potential gains in body mass, which might reduce relative PO (W⋅kg^–1^) and thus hinder performance (Mujika et al., 2016). In this regard, the percentage increase in total body mass was very similar with the two interventions – and below 2.0% in both cases – which is in line with the results of previous research, showing a 1–3% increase in cyclists’ body mass after TRT interventions (Bastiaans et al., 2001; Rønnestad et al., 2010, 2011; Vikmoen et al., 2016; Beattie et al., 2017). It is also worth noting that besides improving time trial performance when expressed in absolute values (W), VBRT tended to improve performance relative to body mass (W⋅kg^–1^, *p* = 0.080).

Our study does have some limitations, such as the low sample size (*n* = 17 total) or the relatively short duration of the intervention (6 weeks) – although the latter can actually support the notion that RT induces beneficial adaptations even in the short term. In addition, the fact that subjects had no previous experience with VBRT could have potentially confounded our results, although we tried to minimize this issue by having the participants perform a 2-week familiarization phase before the intervention. Importantly, we did not include a control group performing endurance training alone, which precludes us from drawing strong conclusions on the effectiveness of the analyzed interventions. On the other hand, participants did not perform the tests in the same phase of the menstrual cycle, which could potentially influence the results although the magnitude of these effects seems to be trivial (McNulty et al., 2020). Finally, it must be noted that the 1RM was estimated and not directly measured, thus resulting in a lower accuracy. Further research is therefore needed to confirm the practical relevance of the observed improvements on strength/power indicators. Finally, it is unlikely that our results had been affected by an eventual menstrual cycle effect since the prevalence of amenorrhea/dysmenorrhea was comparable between the two groups and in fact all participants completed the study with an adherence to the intervention sessions of 100%. In turn, some strengths must also be acknowledged, such as the high fitness level of the participants (i.e., highly trained female cyclists competing at least at national level), the novelty of applying VBRT in cyclists, the individual supervision of all TRT/VBRT sessions, the quantification of training loads during both TRT/VBRT and cycling sessions, and the variety of outcomes included (e.g., body composition determined with DXA, assessment of 1RM and MMP on different exercises, as well as assessment of endurance performance through both incremental exercise testing and a simulated time trial).

The present study suggests that the addition of a short-term (6 weeks, 12 sessions in total) intervention consisting of either VBRT or TRT (with similar external or internal training loads in both cases) to the usual endurance program of competitive female cyclists results in a marked improvement in muscle strength/power as well as in a slight increase in time trial performance (∼3 to 5%), with no differences between interventions but with VBRT inducing greater increases in maximum strength/power for the hip thrust exercise. From a practical point of view, these results might encourage cyclists to implement resistance training interventions, being able to choose either VBRT or TRT depending on their individual preferences or methodological resources. Further research is warranted to confirm the benefits of these two types of interventions compared to a control group performing endurance training alone, as well as to assess if VBRT could provide additional benefits in the longer term.

## Data Availability Statement

The raw data supporting the conclusions of this article will be made available by the authors, without undue reservation.

## Ethics Statement

The studies involving human participants were reviewed and approved by the Hospital Universitario Fundación Alcorcón (19/86). The patients/participants provided their written informed consent to participate in this study.

## Author Contributions

PV, DB-G, AL, and AM-P contributed to the conception and design of the experiments. AM-P, DB-G, LA, JG-C, and ET involved in pretesting, experimental preparation, and data collection. AM-P, DB-G, LA, PV, JG-C, ET, and AL contributed to the analysis and interpretation. AM-P, DB-G, LA, PV, and AL wrote the first version of the manuscript. All authors read and approved the final version of the manuscript.

## Conflict of Interest

The authors declare that the research was conducted in the absence of any commercial or financial relationships that could be construed as a potential conflict of interest.
